# CHIP stabilizes amyloid precursor protein via proteasomal degradation and p53-mediated trans-repression of β-secretase

**DOI:** 10.1111/acel.12335

**Published:** 2015-03-13

**Authors:** Amir Kumar Singh, Uttam Pati

**Affiliations:** School of Biotechnology, Jawaharlal Nehru UniversityNew Delhi, 110067, India

**Keywords:** Alzheimer’s disease, Neurodegenerative diseases, p53, proteasome, ubiquitin pathway

## Abstract

In patient with Alzheimer’s disease (AD), deposition of amyloid-beta Aβ, a proteolytic cleavage of amyloid precursor protein (APP) by β-secretase/BACE1, forms senile plaque in the brain. BACE1 activation is caused due to oxidative stresses and dysfunction of ubiquitin–proteasome system (UPS), which is linked to p53 inactivation. As partial suppression of BACE1 attenuates Aβ generation and AD-related pathology, it might be an ideal target for AD treatment. We have shown that both in neurons and in HEK-APP cells, BACE1 is a new substrate of E3-ligase CHIP and an inverse relation exists between CHIP and BACE1 level. CHIP inhibits ectopic BACE1 level by promoting its ubiquitination and proteasomal degradation, thus reducing APP processing; it stabilizes APP in neurons, thus reducing Aβ. CHIP^U^^box^ domain physically interacts with BACE1; however, both U-box and TPR domain are essential for ubiquitination and degradation of BACE1. Further, BACE1 is a downstream target of p53 and overexpression of p53 decreases BACE1 level. In HEK-APP cells, CHIP is shown to negatively regulate BACE1 promoter through stabilization of p53’s DNA-binding conformation and its binding upon 5′ UTR element (+127 to +150). We have thus discovered that CHIP regulates p53-mediated trans-repression of BACE1 at both transcriptional and post-translational level. We propose that a CHIP–BACE1–p53 feedback loop might control APP stabilization, which could further be utilized for new therapeutic intervention in AD.

## Introduction

Deposition of amyloid-beta (Aβ) as a form of senile plaque in the human brain is a major pathological feature of Alzheimer’s disease (AD) (Murphy & LeVine, 2010). Aβ is produced from the amyloid precursor protein (APP) through sequential proteolytic cleavages by β- and γ-secretases (Li *et al*., 2006). Proteolytic cleavage of APP at the β-site is a rate-limiting step for Aβ generation; β-site APP-cleaving enzyme 1 (BACE1) is the β-secretase *in vivo* (Cai *et al*., 2001). Although the large majority of AD cases are sporadic, autosomal-dominant mutations in APP and presenilin genes might also be responsible for the rare, familial form (Gatz *et al*., 2006).

Oxidative stress has long been implicated in the pathogenesis of AD and is potential cause of the increased level and activity of BACE1 (Tamagno *et al*., 2002, 2008; Tong *et al*., 2005). Multiple studies have explained the molecular mechanism underlying oxidative stress-induced BACE1 activation that might be contributed by redox-sensitive transcriptional factors such as NF-kβ (Bourne *et al*., 2007), HIF (Sun *et al*., 2006; Zhang *et al*., 2007), STAT1 (Cho *et al*., 2009), PPARγ (Sastre *et al.,* 2006), YY1 (Nowak *et al*., 2006), Sp1 (Christensen *et al*., 2004), and dysfunction of ubiquitin–proteasome system (UPS) (Upadhya & Hegde, 2007; Riederer *et al*., 2011). Various oxidative stress markers are found to be elevated in the AD brain, such as HNE, 3-NT, protein carbonyls (Cenini *et al*., 2008), and are associated with p53 tumor suppressor protein that can change its conformation, resulting in loss of DNA binding as well as transcriptional activity (Uberti *et al.,* 2006). In response to oxidative stress, p53, a key regulator, is activated and transactivates its target genes through binding upon specific DNA sequences in their promoter region (Gambino *et al*., 2013). An increase in the expression of p53 and its altered conformation have been observed in brain and peripheral cells of patients with AD, leading to intense dysfunction in the p53 signaling pathway in response to various stresses, without any evidence of genetic mutations (Uberti *et al*., 2006; Lanni *et al*., 2007, 2008). While there is an increase in the total p53 level, the level of phosphorylated p53-Ser^15^ decreased along with a decrease in the total p21 and phosphorylated p21-Thr^145^ level in peripheral blood lymphocytes of patients with AD (Tan & Evin, 2012), suggestive of G1/S check point dysfunction (Zhou & Jia, 2010).

The levels and activity of BACE1 increased in the brain of patients with sporadic AD (Yang *et al*., 2003) as BACE1-knockout mice lack Aβ generation and are free from AD-associated pathologies (Luo *et al*., 2001). It is suggested that dysfunction of UPS might also be involved in AD pathogenesis (Upadhya & Hegde, 2007; Riederer *et al*., 2011). BACE1 proteins are degraded through the UPS (Qing *et al*., 2004) in which brain-specific SFC E3-ligase helps in recognition, ubiquitination, and degradation coinciding with decrease in the production of Aβ (Gong *et al*., 2010). Interestingly, the accumulation of Aβ is also shown to activate BACE1 expression (Piccini *et al*., 2012) along with a decrease in activity of UPS (Almeida *et al*., 2006). Thus, partial suppression of BACE1 attenuates Aβ generation and AD-related pathology (Kimura *et al*., 2010), suggesting that partial inhibition of BACE1 could be an ideal target for AD treatment.

We had earlier shown that CHIP, a brain-enriched E3 ligase, chaperones p53 and stabilizes its native conformation under stress (Tripathi *et al*., 2007). It further contributes to ubiquitination and degradation of several AD-related proteins, such as CFTR (Meacham *et al*., 2001), tau (Petrucelli *et al*., 2004), p53 (Esser *et al*., 2005), APP, and Aβ (Kumar *et al*., 2007). Its expression was shown to decrease upon Aβ accumulation in both transgenic mice and cultured cells (Oddo *et al*., 2008). As the increased activity of BACE1 and increased expression of p53 with altered conformation are observed in brain of patients with sporadic AD, we hypothesized whether CHIP would destabilize BACE1 via proteasomal degradation and whether it could stabilize p53 to trans-repress BACE1 gene transcription. In this report, we have shown in neurons that CHIP decreases BACE1 protein level, thus stabilizing APP in reducing Aβ production. CHIP promotes ubiquitination and proteasomal degradation of BACE1 and CHIP-mediated p53 stabilization results in negative regulation of BACE1 gene transcription. Hence, CHIP’s function in stabilizing APP at both transcription and post-translational level might open up new strategies in therapeutic control of AD.

## Results

### CHIP prevents β-cleavage of APP through BACE1 destabilization

**Fig 1:**
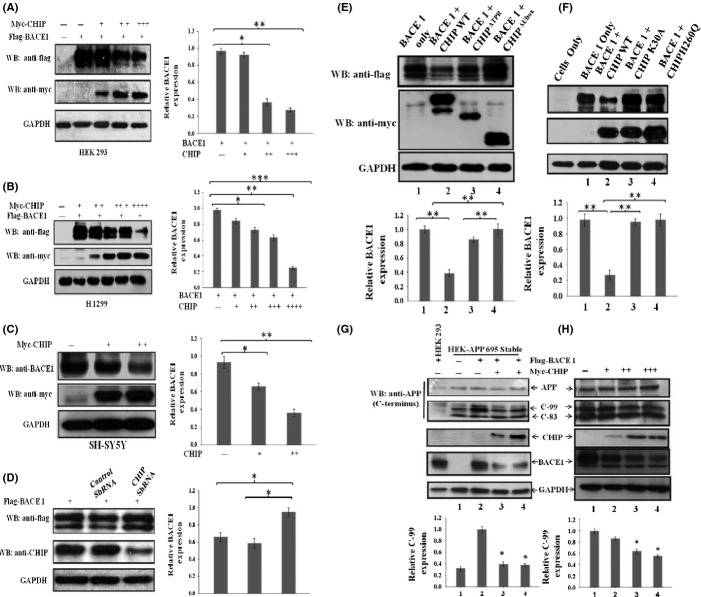
CHIP prevents β-cleavage of APP through BACE1 destabilization. (A–B) Destabilization of ectopic BACE1 by CHIP (0.5, 1, 2, and 3) μg in a dose-dependent manner. (C) CHIP destabilizes endogenous BACE1 level in SH-SY5Y neuroblastoma cells. (D) Silencing of endogenous CHIP stabilizes BACE1 protein level. (E) and (F) Functional domain of CHIP responsible for BACE1 destabilization. (G–H) CHIP destabilizes BACE1 protein level and decreases APP processing at β-cleavage site. (G) HEK 293 cell stably expressing APP were co-transfected with Flag-BACE1 constructs along with increasing amounts of myc-CHIP constructs. (H) Rat primary cortical neurons were transfected with increasing amount of myc-CHIP. After 30 h of transfection, C-terminal β-site cleavage product of APP in whole-cell lysate was determined by Western blotting using anti-APP antibodies (C-terminal). All the data were expressed as mean ±SE from three independent experiments. Statistical analysis were performed by one-group *t-*test for the significance at the * = *P *< 0.05, ** = *P *< 0.01 and *** =*P *< 0.001.

CHIP, a chaperone-associated E3-ligase, regulates the stability of several diseases-associated brain proteins through the ubiquitin–proteasome pathway. Although CHIP stabilizes APP, it also helps in the ubiquitination of APP that are destined for proteasome degradation. During AD pathogenesis, the levels of CHIP decrease along with an increase in the expression of BACE1, which is degraded through UPS (Qing *et al*., 2004). To determine the role of CHIP in the regulation of BACE1 during AD pathogenesis, we first asked whether BACE1 is a substrate for CHIP and checked the stability of BACE1 in the presence of CHIP. We expressed Flag-BACE1 alone or in the presence of increasing amounts of myc-CHIP in human HEK 293 and H1299 cells and estimated the level of BACE1 and CHIP by Western blotting. We observed that the ectopic expression of BACE1 was significantly reduced by CHIP in a dose-dependent manner as compared to BACE1 when expressed alone (Fig.1A,B). Further, endogenous expression of BACE1 protein was also reduced by CHIP in human neuroblastoma cells (SH-SY5Y) (Fig.1C). Next, we investigated whether endogenous CHIP can directly modulate the stability of BACE1 protein. The silencing of endogenous CHIP by shRNA resulted in the stabilization of BACE1 protein as compared to control where BACE1 was transfected with either scrambled shRNA or empty plasmid (Fig.1D). This result establishes that CHIP negatively regulates BACE1 stability.

CHIP contains an N-terminal TPR domain that is responsible for chaperone binding, a C-terminal U-box domain possessing E3-ubiquitin ligase activity and a central charged domain. To identify the CHIP domains that regulate BACE1 stability, a series of CHIP mutants were examined for their abilities to regulate BACE1 stability. Both TPR and U-box deletion constructs of CHIP were unable to reduce BACE1 protein level (Fig.1E). Furthermore, point mutations K30A (TPR domain) and H260Q (U-box domain), which abolish the ability of CHIP to interact with chaperones and of its ubiquitin ligase activity, respectively, also failed to reduce BACE1 protein (Fig.1F), thus suggesting that both TPR and U-box domains of CHIP are necessary for BACE1 degradation.

We further investigated the role of CHIP in the β-cleavage of APP and Aβ generation in HEK-APP stable cells and rat primary cortical neurons. HEK 293 cells stably expressing human APP695 were transfected with cDNA encoding Flag-BACE1 along with control plasmid or with myc-CHIP, and the levels of BACE1 cleavage product of APP (CTFβ 99) and other proteins were estimated by Western blotting. Further, rat primary cortical neurons were transfected with control plasmid or with increasing amount of myc-CHIP and the level of endogenous BACE1, CTFβ 99 (C-99), and other proteins were estimated. The results show that CHIP significantly reduced BACE1 cleavage product of APP (CTFβ 99) as compared to control plasmid in both HEK-APP cells (Fig.1G) and rat primary cortical neurons (Fig.1H). Thus, CHIP destabilizes BACE1 protein and decreases APP processing at the β-secretase site to attenuate Aβ generation.

### CHIP promotes BACE1 ubiquitination and proteasomal degradation

**Fig 2:**
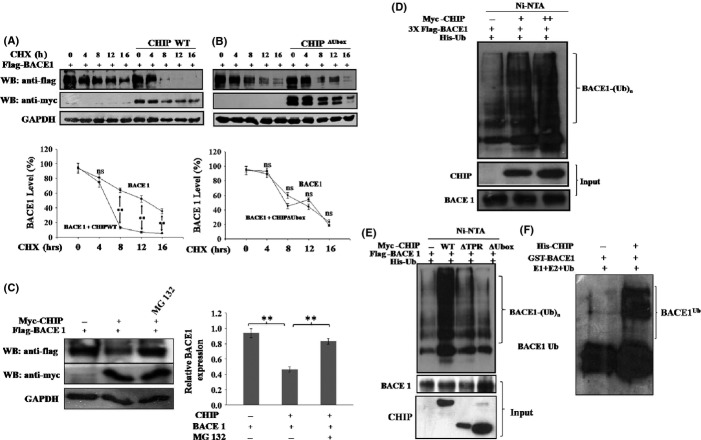
CHIP promotes BACE1 ubiquitination and proteasomal degradation. (A–B) Destabilization of BACE1 at post-translational level by CHIP was determined by cycloheximide chase assay. HEK 293 cells were transfected with Flag-BACE1 along with myc-CHIP (A) or myc-CHIP^ΔUbox^ (B). After 20 h of transfection, cells were treated with 100 μg/mL of cycloheximide (CHX) at indicated time points to inhibit protein synthesis. (C) Proteasomal-dependent degradation of BACE1 by CHIP. HEK 293 cells were co-transfected with Flag-BACE1 along with myc-CHIP. After 24 h of transfection, cells were treated with 20 μm MG12 for 6 h to inhibit proteasome activity. (D–E) CHIP promotes BACE1 ubiquitination. HEK 293 cells were co-transfected with Flag-BACE1 and His-ubiquitin (His-Ub) in the presence of an increasing amount of CHIP (D) or its deleted mutants (E). Ubiquitinated BACE1 from whole-cell lysate was precipitated with Ni^2+^-NTA beads followed by Western blotting with anti-flag antibodies. (F) GST-BACE1 (2 μg) was incubated with E1 and E2 (UbcH5a) and ubiquitin (2 μm) in the presence or absence of His-CHIP (5 μg) for 30 min. Ubiquitination of BACE1 was analyzed by Western blotting using anti-BACE1 antibodies. All the data were expressed as mean ±SE from three independent experiments. Statistical analysis were performed by one-group *t-*test for the significance at the ** = *P *< 0.01. ns, nonsignificant.

To check whether the reduced stability of BACE1 by CHIP is a post-translational event, we first examined half-life of BACE1 protein using the protein synthesis inhibitor cycloheximide (CHX). HEK 293 cells were co-transfected with BACE1 cDNA along with empty vector, myc-CHIP, or myc-CHIP^ΔUbox^. The rate of degradation of BACE1 was found to be significantly faster in the presence of CHIP as compared to BACE1 expressed alone or in the presence of CHIP^ΔUbox^, which lacks E3-ligase activity (Fig.2A,B). Therefore, CHIP promotes destabilization of BACE1 at the post-translational level and its E3-ligase activity was essential for BACE1 degradation. CHIP-mediated destabilization of the BACE1 at post-translational level led us to investigate the mechanism through which CHIP exerts its effect upon BACE1 protein. Previous studies had established the role of CHIP in promoting proteasome-dependent degradation of its client proteins, such as hTERT (Lee *et al*., 2010), PTEN (Ahmed *et al*., 2012), and p53 (Esser *et al*., 2005). The turnover of BACE1 protein is also controlled through a proteasome-dependent pathway (Qing *et al*., 2004; Gong *et al*., 2010). The treatment of cells with MG132, an inhibitor of proteasome, caused stabilization of BACE1, and the level of BACE1 that was decreased by co-expression of CHIP was reverted back to normal (Fig.2C**)**. This result clearly indicates that the treatment of cells with proteasome inhibitor rescued BACE1 from CHIP-induced destabilization, thus establishing that CHIP promoted destabilization of the BACE1 protein through the proteasome-mediated degradation. We then performed ubiquitination assay for BACE1 protein with CHIP. HEK 293 cells were transfected with Flag-BACE1 and 6X His-ubiquitin (His-Ub) in the presence of CHIP or empty plasmid. Cells were lysed and washed under denaturing conditions to ensure both inactivation of deubiquitinating enzymes and removal of contaminating proteins, except proteins that are covalently associated with Ub. Proteins that were conjugated with His-Ub were pulled down by Ni^2+^-NTA beads followed by Western blotting with anti-flag antibodies to detect ubiquitin-conjugated BACE1. Western blot results showed that high molecular weight ubiquitinated forms of BACE1 were considerably enhanced when BACE1 was co-expressed along with CHIP in a dose-dependent manner (Fig.2D), suggesting that CHIP was responsible for BACE1 ubiquitination. The ubiquitination of BACE1 was also examined in the presence of CHIP mutants. It was observed that only wild-type CHIP enhanced the ubiquitination of BACE1, while the deletion mutants CHIP^ΔTPR^ and CHIP^ΔUbox^ failed to ubiquitinate BACE1 (Fig.2E). The ubiquitination level of BACE1 in the presence of CHIP deletion mutants was similar as it was observed in the presence of an empty plasmid. Thus, both deletion mutants of CHIP could not enhance the ubiquitination of BACE1, suggesting that both domains of CHIP were essential for ubiquitination and degradation of BACE1. Further, *in vitro* ubiquitination reaction was performed to confirm that CHIP is directly involved in the ubiquitination of BACE1 (Fig.2F).

### BACE1 physically interacts with CHIP’s U-box domain

**Fig 3:**
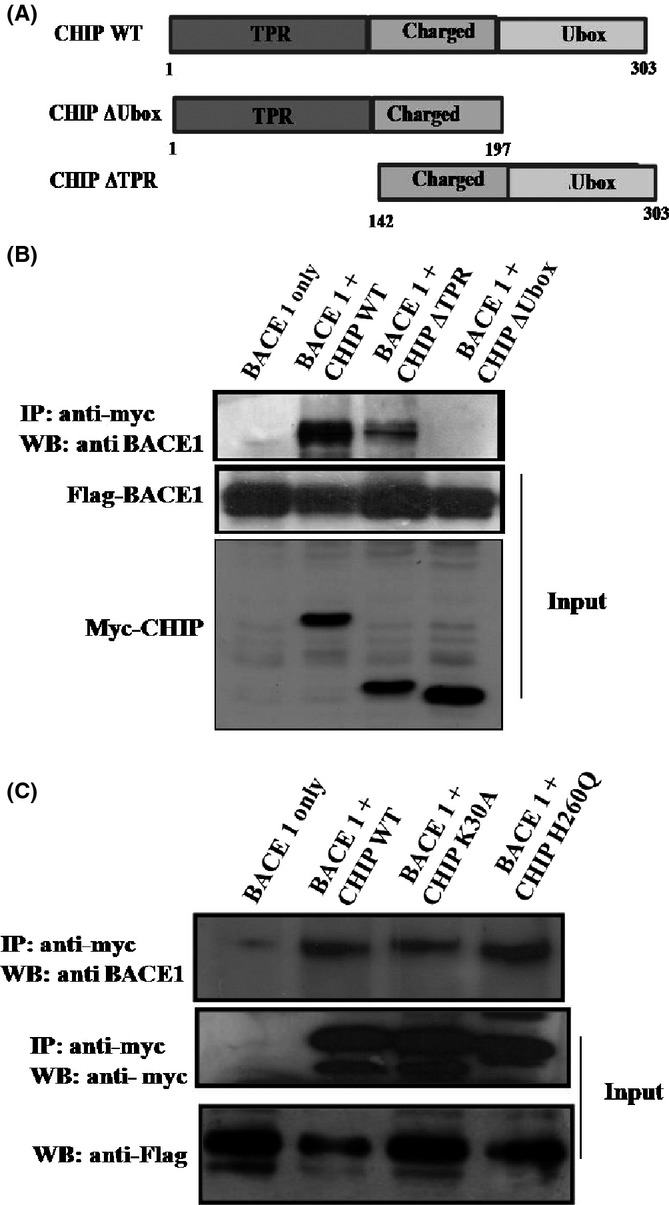
BACE1 physically interacts with CHIP. (A) Schematic representation of CHIP and its deleted mutants used in the study. (B) Interaction between BACE1 and CHIP mutants. Two micrograms of Flag-BACE1 was co-transfected with 2 μg of myc-CHIP wild-type or its mutant. Co-immunoprecipitation assay was performed using anti-myc antibodies against CHIP, followed by Western blotting with anti-flag antibodies against the BACE1. BACE1 interacts with CHIP through its U-box domain. (C) Interaction between CHIP and BACE1 was independent of point mutations.

As BACE1 is shown to be a natural substrate of CHIP, we then examined the physical interaction between CHIP and BACE1. We first performed a co-immunoprecipitation assay in HEK 293 cells where Flag-BACE1 was co-transfected with myc-CHIP or its deletion mutants (Fig.3A). The deletion mutant CHIP^ΔTPR^ lacks the TPR domain (1–141 aa) and is defective in chaperone binding ability, whereas the CHIP^ΔUbox^ lacks U-box domain (198–303 aa) and is defective in E3-ligase activity. We found that CHIP physically associates with BACE1 through its U-box domain, as assessed by co-immunoprecipitation with anti-myc antibodies followed by Western blotting detection with anti-flag antibodies**.** Both CHIP and CHIP^ΔTPR^ were co-immunoprecipitated with BACE1, whereas CHIP^ΔUbox^ failed to co-immunoprecipitate with BACE1**,** revealing that U-box domain of CHIP interacts with BACE1 (Fig.3B).

Several studies have reported that the association of CHIP with Hsp70/90 was important for its interaction with client proteins through TPR domain (Lee *et al*., 2010; Ahmed *et al*., 2012). To check whether interaction between CHIP and BACE1 is mediated through Hsp70/90, we expressed Flag-BACE1 along with myc-CHIP, myc-CHIP^K30A^, or myc-CHIP^H260Q^ mutants in HEK 293 cells. Substitution mutation K30A in the TPR domain of CHIP prevented its interaction with Hsp70/90. H260Q mutation in the U-box domain of CHIP also prevented the interaction of CHIP with E2 enzymes and results in loss of its E3 ligase activity. Co-immunoprecipitation with anti-myc antibodies followed by Western blotting with anti-flag antibodies showed that BACE1 co-immunoprecipitates with CHIP, CHIP^K30A^, and CHIP^H260Q^ mutants (Fig.3C). These results implied that the interaction between BACE1 and CHIP is independent of Hsp70/90.

### Transcriptional repression of BACE1 by p53

**Fig 4:**
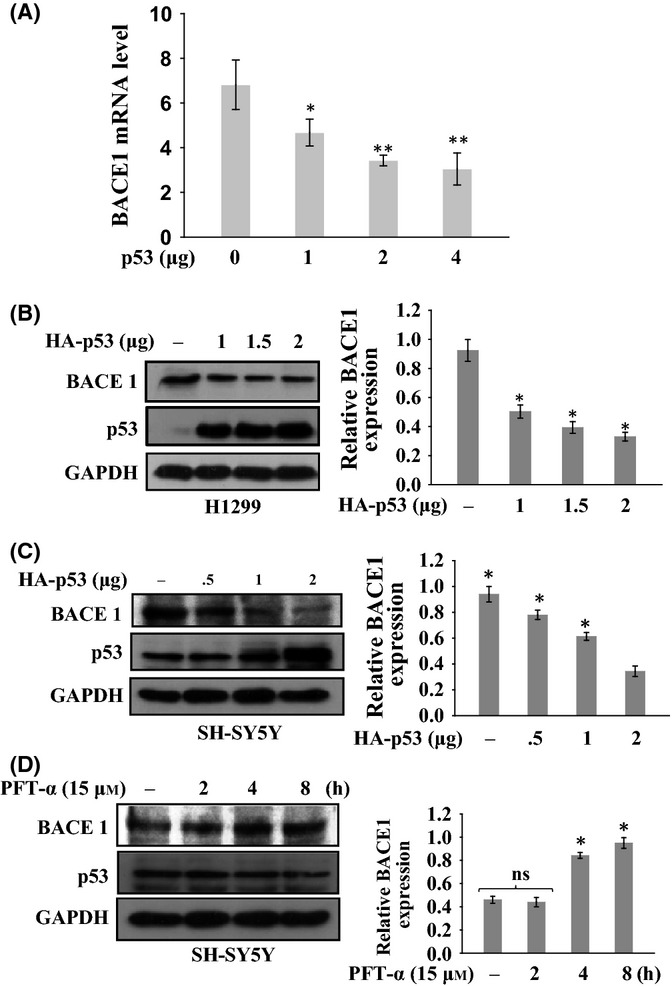
Transcriptional repression of BACE1 by p53. (A) Effect of p53 on BACE1 mRNA level. SH-SY5Y cells were transfected with increasing amount (1, 2, and 3 μg) of p53. Quantitative real-time PCR analysis was performed. The endogenous BACE1 mRNA level decreases with increasing p53 expression. (B–C) Effect of p53 on BACE1 protein level. (A) H1299 and (C) SH-SY5Y cells were transfected with increasing amount (1, 1.5, and 2 μg) of p53 expression constructs. (D) Effect of inhibited endogenous p53 on BACE1 level. SH-SY5Y cells were treated with PFT-α (15 μm) for indicated time period to inhibit endogenous p53. Endogenous BACE1 protein levels were determined by Western blotting using anti-BACE1 antibodies. Data represent the mean ± SE from three independent experiments. Statistical analysis were performed by one-group *t-*test for the significance at the *=*P *< .005 and **=*P *< 0.001. ns, nonsignificant.

The expression of BACE1 is tightly regulated at the transcriptional level and its dysregulation leads to its overexpression during AD pathogenesis (Sun *et al*., 2012). Enhanced transcription of BACE1 in patients with AD was shown to be synchronized with functional inactivation of p53 (Uberti *et al*., 2006; Lanni *et al*., 2007, 2008) and that led us to ask whether p53 negatively regulates BACE1 gene transcription. SH-SY5Y cells were transfected with increasing dose of p53 followed by quantitative real-time PCR (qPCR) and Western blotting analysis. The cDNA synthesis was carried out from total RNA, and qPCR was performed in triplicate using cDNA. Relative BACE1 mRNA expression level was normalized to an endogenous housekeeping gene (GAPDH). The result showed that exogenous expression of p53 decreases the expression of BACE1 at mRNA level in a dose-dependent manner (Fig.4A). This result confirmed that p53 suppresses BACE1 expression at the transcriptional level.

As BACE1 transcription is downregulated by p53, as revealed by mRNA level of BACE1, we then investigated the effects of p53 on the BACE1 protein level. We transfected H1299 cells (p53^−/−^) and SH-SY5Y cells (p53^+/+^) with increasing amount of p53 to examine the effect of overexpression of p53 on the endogenous BACE1 level. As the expression of p53 increased, the level of endogenous BACE1 protein was decreased in a dose-dependent manner in both cell lines (Fig.4B,C). Inhibition of p53 expression by pifithrin-α in SH-SY5Y cells showed that BACE1 protein level was increased with increasing duration of treatment (Fig.4D). This clearly indicated that the p53 downregulates the expression of BACE1 protein.

### p53 downregulates BACE1 promoter through selective binding at +127 to +150 position

**Fig 5:**
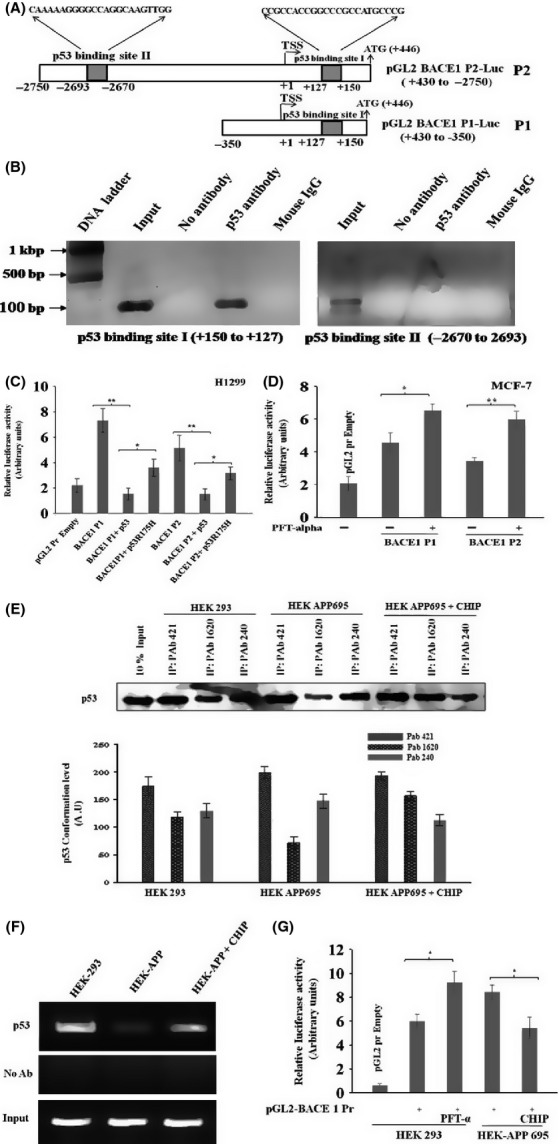
p53 selectively binds to BACE1 promoter. (A). Schematic representation of BACE1 promoter. Boxes shown are the two potential p53 binding sites in the BACE1 promoter, identified by MatInspector software. (B) ChIP assay shows that p53 binding site on BACE1 promoter. H1299 cells were transfected with p53 expression plasmid. Cells were fixed and DNA was precipitated with anti-p53 antibodies followed by PCR amplification. (C) Luciferase reporter plasmids carrying the *BACE1* promoter regions were co-transfected with wild-type p53 and mutant p53 into H1299 cells. (D) The p53 inhibitor PFT-α prevents the repression of BACE1 transcriptional activity in MCF-7 (p53^+/+^) cells. Cells were transfected with plasmid carrying the *BACE1* promoter region and treated with PFT-α (15 μm, 6 h) to inhibit endogenous p53. (E–F) Downregulation of BACE1 is due to CHIP-mediated p53 stabilization. (E) HEK-APP stable cells were transfected with CHIP expression plasmids. p53 was immunoprecipitated with conformation-specific antibodies PAb 1620 (wild-type) and PAb 240 (mutant) to analyze the conformational state of p53 followed by Western blotting with anti-p53 (FL-393) antibodies. (F) ChIP assay were performed with anti-p53 antibodies (FL-293) on HEK 293 and HEK-APP cells. PCRs were performed on the immunoprecipitated DNA samples using specific primers for the BACE1 promoter. A sample representing linear amplification of the total input chromatin (Input) was included as control. Additional controls included immunoprecipitation performed with nonspecific immunoglobulins (no Ab). (G) Luciferase reporter plasmids carrying the *BACE1* promoter regions were transfected alone or with CHIP in to HEK 293 and HEK-APP cells. Cells were treated with PFT-α (15 μm) for next 6 h. Relative luciferase activity was determined in triplicate, and β-galactosidase is used for normalization of transfection efficiency. Data represent the mean ± SE from three independent experiments. Statistical analysis were performed by one-group *t-*test for the significance at the *=*P *< .005 and **=*P *< 0.001.

As p53 negatively regulates BACE1 expression, we searched for p53 binding sites upon BACE1 promoter through *in silico* analysis using MatInspector tool (Cartharius *et al*., 2005). Two sites were found: the first one was located downstream of the start site (TSS) at (+150 to +127, score: 0.942), whereas the second one was at (−2670 to −2693, score: 0.847) (Fig5A). Chromatin immunoprecipitation (ChIP) assay was performed in H1299 cells that were transfected with p53 cDNA. Anti-p53 antibodies were used for immunoprecipitation followed by PCR with flanking primers for both p53DBS. Amplification of a 182-bp region of putative p53 binding site I (+150 to +127) (Fig.5B) was observed; no band was observed with primers for the site II at (−2570 to −2693). Similar results were observed with controls that contain no antibodies or nonspecific antibodies; PCR with input sample showed amplification against both the regions (Fig.5B). This result clearly shows that p53 exclusively binds to the BACE1 promoter in the region of 5′ UTR (+150 to +127 bp). In some genes, 5′ UTR is a part of core promoter that contains transcriptional factor binding sites and regulates its transcription (Yu *et al*., 2001). This result indicates that p53 transcriptional factor directly binds to the BACE1 promoter at the site of +150 to +127 (5′ UTR) and not upon the site of −2670 to −2693. The effect of p53 binding upon DBS of the 5′ UTR in the BACE1 promoter was then analyzed by luciferase assay. H1299 (p53^−/−^) cells were transfected with either the pGL2 BACE1 P1-Luc (P1) or pGL2 BACE1 P2-Luc (P2) (Fig5A) along with the p53 expression plasmid. The co-transfection of BACE1 promoter constructs along with p53 resulted in significant repression (5-fold) of BACE1 promoter activity (Fig.5C). By contrast, co-transfection of pGL2 BACE1 promoter constructs along with p53 R175H, a p53 mutant that does not bind to DNA, resulted in partial inhibition of the BACE1 promoter activity as compared with wild-type p53 (Fig.5C). Transcriptional repression ability of mutant p53 might not depend exclusively on functional sequence-specific DNA-binding domain of p53. An earlier study shows that p53 R175H mutant exerted repression activity on *MAD1* promoter with weaker DNA-binding activity as compared to wild-type p53 (Chun & Jin, 2003). An inactivation of endogenous p53 by pifithrin-α (PFT-α) in MCF-7 (p53^+/+^) cells caused activation of BACE1 promoter activity as compared to untreated cells (Fig.5D). These findings clearly demonstrate that p53 interacts with BACE1 promoter and downregulates its transcriptional activity.

### CHIP-mediated p53 stabilization downregulates BACE1 gene promoter

Previous studies have reported that HEK-APP stable cells have high levels of oxidative stress markers and unfolded p53 conformation, which may be due to its nitration at tyrosine residues. It is assumed that unfolded p53 is responsible for the loss of its transcriptional activity and reduced sensitivity to various cytotoxic insults (Uberti *et al*., 2007; Buizza *et al*., 2013). The expression level of CHIP increases significantly in cells upon exposure to various stresses and this could be an adaptive response of the cell to deal with the excess burden of misfolded protein (Dikshit & Jana, 2007). CHIP also protects folded p53 conformation and restores its DNA-binding and transcription activity (Tripathi *et al*., 2007). To investigate whether CHIP can stabilize the folded p53 conformation and restores its transcriptional activity in HEK-APP stable cells, the effect of CHIP on p53 conformation was analyzed. For this, HEK-APP cells were transfected with CHIP expression plasmid and cell lysates were immunoprecipitated using two p53 conformation-specific antibodies, PAb1620 (folded p53) and PAb240 (unfolded p53). The level of unfolded p53 conformation is high (PAb 240) in HEK-APP cells (Fig.5E) as compared to mock HEK 293 cells. However, when HEK-APP cells were transfected with CHIP, the folded conformation (PAb 1620) of p53 was restored and at the same time, a decrease in the expression of unfolded p53 was observed. This result clearly demonstrates that CHIP protects folded p53 conformation, which was getting unfolded due to APP metabolism in HEK-APP cell. Further, we investigated whether p53 binding upon target promoters is somehow compromised in HEK-APP cells. As shown in Fig.5F, ChIP assay shows that the p53 recruitment onto BACE1 promoter was present in HEK 293 cell, whereas it was reduced in HEK-APP cells. Of note, upon transfection of CHIP to HEK-APP restored p53 binding activity to DNA, likely counteracting the misfolding of p53 confirmation and thus suggesting that CHIP was able to restored the DNA-binding activity of p53. Next, we assessed the transcription activity of BACE1 in HEK-APP cells. We transfected the plasmid carrying BACE1 promoter into HEK-APP and HEK 293 cells, and transcriptional activity of p53 was analyzed. As it was expected, in mock HEK 293 cells, inactivation of p53 by treating with PFT-α increased BACE1 promoter activity as compared to untreated cells (Fig.5G). But, in HEK-APP stable cells, BACE1 promoter activity is increased corresponding to mock HEK 293 cells that were treated with PFT-α. However, exogenous expression of CHIP in HEK-APP cells decreased BACE1 promoter activity (Fig.5G). This result shows that CHIP protects p53 transcriptional activity in HEK-APP cells and represses BACE1 promoter activity.

## Discussion

**Fig 6:**
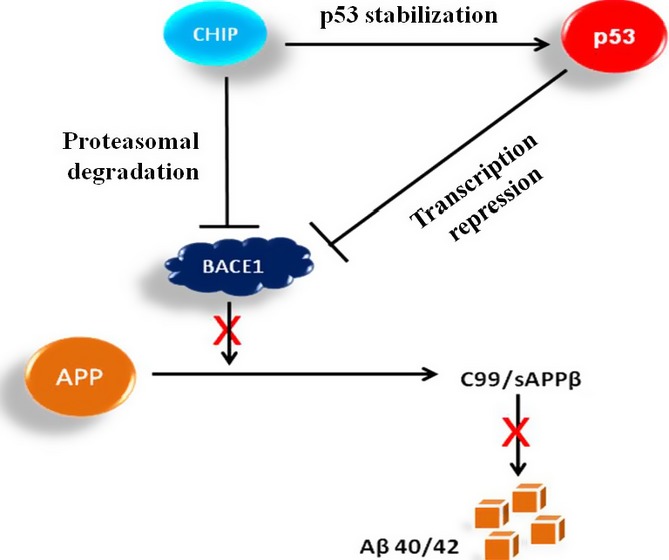
CHIP-mediated p53 stabilization prevents APP degradation. Model proposing that a CHIP–p53–BACE1 loop might exist in AD.

BACE1 plays a central role in the AD pathogenesis by processing APP to Aβ (Cai *et al*., 2001). Therefore, understanding the mechanism of regulation of APP and BACE1 is critical for designing therapeutic strategies for AD. In this study, we have shown that BACE1 is a new substrate of CHIP which binds to CHIP’s U-box domain and CHIP promotes BACE1 destabilization through UPS by promoting its ubiquitination (Fig.6). CHIP and HSPs were earlier shown to interact with β-APP in a proteasome-dependent manner and influence Aβ metabolism (Kumar *et al*., 2007). We have also demonstrated the functional consequence of BACE1 regulation by CHIP on APP processing in both neurons and HEK-APP stable cells in which overexpression of CHIP reduced BACE1 activity through decreased β-cleavage product (CTFβ-99) and Aβ generation. Recently, it was reported that Aβ induces BACE1 expression in primary astrocytes as well as in human astrocytoma cell line (Piccini *et al*., 2012; Tan & Evin, 2012). One might thus presume that an increased level of BACE1 during AD pathogenesis could be due to decreased expression of CHIP, which results in CHIP-mediated BACE1 degradation.

An impairment of proteasome activity was earlier reported in patients with AD which could be a possible reason for the elevation of BACE1 during AD pathogenesis (Upadhya & Hegde, 2007). Moreover, another E3-ligase complex SFC^Fbx2^ that degrades BACE1 through proteasome was found to be reduced in patients with AD and AD mouse model (Gong *et al*., 2010). Thus, there might exist an inverse relation between CHIP and BACE1. Recently, it was also reported that mutant ubiquitin B (UBB+1) was found in the brain of patients with AD (Fischer *et al*., 2003), thus causing attenuation of proteasome activity along with reduced BACE1 degradation (Zhang *et al*., 2010). Although interaction of CHIP with Hsp70/90 helps in recognizing new substrates and plays an important role in the client protein regulation, CHIP physically interacts with substrates through either its TPR or U-box domain. Our results show that BACE1 interact with CHIP’s U-box domain and this interaction is independent of molecular chaperones Hsp70/90. In a similar manner, highly charged region and U-box domain of CHIP interacts with interferon regulatory factor-1 (IRF-1), indicating no role of chaperones in this interaction (Narayan *et al*., 2011). Ubiquitination and degradation of Tal1/SCL are induced by notch signaling and depend on Skp2 and U-box domain (Nie *et al*., 2008). By contrast, CHIP interacts with RunX1, a member of the Runt transcription factor, through its TPR and charged domain and with interferon regulatory factor-1 (IRF-1) (Shang *et al*., 2009).

During progression of AD, in the brain of patients with AD, oxidative stress markers associate with p53, leading it into unfolded conformation and impaired transcriptional activity (Lanni *et al*., 2007; Cenini *et al*., 2008; Zhou & Jia, 2010). These unfolded p53 increases APP metabolism and Aβ load and vice versa (Uberti *et al*., 2007; Cai & Ratka, 2011; Buizza *et al*., 2013). Level of CHIP mRNA was shown to be significantly increased in cells exposed to oxidative stress, and it could be an adaptive response to deal with the burden of misfolded proteins (Dikshit & Jana, 2007). By contrast, selective accumulation of Aβ_42_ with a reduction in CHIP expression markedly accelerates the progression of tau pathology, which is rescued by restoring CHIP level (Oddo *et al*., 2008). In HEK-APP stable cells, Aβ changes conformation of p53 to unfolded state through degradation of homeodomain interacting protein kinase 2 (HIPK2) (Lanni *et al*., 2010). We had earlier shown that CHIP preferentially binds to the unfolded p53 conformation and restores its DNA-binding activity, which is independent of Hsp70 (Tripathi *et al*., 2007). But, when CHIP was expressed in HEK-APP cell, the DNA-binding activity of p53 was restored due to chaperone activity of CHIP. This suggests that CHIP might be responsible for partially restoring p53 wild-type conformation, thus enhancing DBS binding upon BACE1 promoter and promoting transcriptional repression.

Furthermore, oxidative stress elevates transcription of *BACE1* gene through redox-sensitive transcription factors (Christensen *et al*., 2004; Sastre *et al*., 2006; Sun *et al*., 2006; Bourne *et al*., 2007; Zhang *et al*., 2007). Our results show that an overexpression of p53 suppresses BACE1 expression via its binding upon BACE1 promoter; the suppression of BACE1 expression by p53 is one of pathways by which p53 might inhibit APP processing and Aβ generation during AD pathogenesis. Interestingly, BACE1 promoter activity is increased in HEK-APP stable cells as compared to HEK 293 cells. Our finding could be correlated by an earlier study which showed that an impairment of p53 signaling pathway and G1/S check point dysfunction was found in patients with AD (Uberti *et al*., 2002; Zhou & Jia, 2010).

In summary, our study shows that BACE1 is a p53 downstream gene and can be downregulated at both transcriptional and post-translational level by CHIP-mediated p53 activation and CHIP-mediated degradation. We purpose that an inverse relation between CHIP and BACE1 through a possible p53–CHIP–BACE1 feedback loop (Fig. 6) might be an indicator during AD pathogenesis; the stabilization of p53 as well as of APP seems to be linked to AD pathogenesis. This study might bring new direction in developing new therapeutic protocols during AD pathogenesis through novel intervention through CHIP–p53–BACE1 loop.

## Experimental procedures

### Cell culture, antibodies, and transfection assay

For luciferase assay, we generated reporter constructs of *BACE1* promoter. About 3.2-kb fragment of the 5′-flanking region (+430 to −2750) of the *BACE1* gene was amplified from the genomic DNA of H1299 cells by PCR using Phusion polymerase. This amplified product was further used as a template for amplification of deletion constructs and cloned into pGL2 promoter vector at *Nhe*I/*Bgl*II site. The 3XFLAG-BACE1 plasmid was generated using specific primers to amplify BACE1 insert and ligated into 3XFLAG-CMV-10 vector at *Hind*III/*Bam*HI restriction site. HA-p53, His-Ub**,** myc-CHIP, myc-CHIP^ΔTPR^, myc-CHIP^ΔUbox^, myc-CHIP^K30A^, and myc-CHIP^H260Q^ were available in our laboratory. H1299, MCF-7, HEK 293, and SH-SY5Y cell lines were obtained from National Centre for Cell Sciences Pune (India). The HEK 293 cells were stably transfected with APP (Uberti *et al*., 2007). The medium used for these cell lines was Dulbecco’s modified Eagle’s medium (DMEM) supplemented with 10% fetal bovine serum. Anti-p53 PAb DO1, FL-393 antibodies (nonconformational), anti-p53 PAb1620 antibodies (wild-type), anti-p53 PAb240 antibodies (mutant conformation), anti-myc antibodies, anti-BACE1 antibodies (Z-183 and M-83), and anti-APP antibodies (c-terminus) were purchased from Santa Cruz, and anti-flag antibodies were from Sigma. All transfections were carried out using Lepofectamine LTX (Invitrogen, USA), except in primary cortical neurons using Lipofectamine Messenger MAX (Invitrogen) according to manufacturer’s instructions. Luciferase assay was performed using luciferase reporter gene assay kit (Promega, USA) according to manufacturer’s instructions. One day before transfection, 2 × 10^5^ cells were seeded in 12-well culture plates, and after 30 h of transfection, the assay was performed. Cells were washed with cold PBS and lysed. Equal volume of luciferin solution and lysate was mixed and luminescence was measured immediately in a luminometer. The experiment was performed in triplicate and data were expressed as mean ± SD.

### Primary cortical neuron culture

Primary cultures of embryonic rat cortical neurons were prepared as described (Kao *et al*., 2004). In brief, dissociated embryonic neurons from pregnant rat were plated onto poly-D-lysine/laminin-coated 35-mm plates and cultured at a density of 4 × 105 cells/plates in a neurobasal medium (Invitrogen) supplemented with B27, L-glutamine, and 1% penicillin–streptomycin sulfate. The neurons were transfected with myc-CHIP using Lipofectamine Messenger MAX. The medium was changed after transfection, and after 48 hrs of transfection, cell lysates were prepared for detecting C99/C89 generation using Western blot analysis.

### Western blot

After protein samples were resolved on SDS-PAGE, proteins were transferred on to the nitrocellulose membrane by wet blot system (Bio-Rad, USA). Post-transfer, membrane was transferred to blocking buffer (PBS, 5% skimmed milk and 0.1% Tween-20) for 1 h at room temperature. After incubation, blot was washed three times (5 min each) with washing buffer (PBS containing 0.1% Tween-20). Subsequently, the membrane was incubated with primary antibodies diluted in PBS with 1% BSA and 0.05% Tween-20 for 1–2 h followed by washing thrice (5 min each) with washing buffer. The membrane was then incubated with secondary antibodies conjugated to poly-horse radish peroxidase (HRP) for another 1 h. After subsequent washing, blot was developed with ECL™ (Millipore, Germany) Western blotting detection reagents. We used GeneSnap/GeneTools software (Syngene, USA) to quantify band density of the blot.

### Immunoprecipitation (IP)

Cells were harvested and lysed in NP-40 buffer (50 mm Tris–HCl, pH 7.4, 150 mm NaCl, 1% NP-40, supplemented with cocktail protease inhibitor) at 4 °C for 20 min. After centrifugation, supernatant was transferred to fresh tubes. Approx 10% of whole-cell lysate was used as input. About 0.5–1 mg of whole-cell lysate was incubated with 1.0 μg of anti-myc antibodies and incubated for 2–3 h at 4 °C. Twenty-five microliters of protein A agarose (50%) was added to the lysate and further incubated at 4 °C for 2 h. Washing was carried out 5 times with NP-40 buffer. Immunocomplex was released by the addition of SDS loading dye, boiled, and analyzed by Western blotting.

### *In vivo* ubiquitination assay

To precipitate ubiquitinated proteins, cells were lysed in 1 mL of buffer I (8 m Urea, 0.1 m Na_2_HPO_4_/NaH_2_PO_4_ pH 8.0, 0.01 m Tris–HCl, pH 8.0, 150 mm NaCl, 0.2% Triton X-100, 20 mm imidazole, and 10 mm β-mercaptoethanol) for 15 m followed by sonication. Fifty microliters of Ni^2+^-NTA-agarose beads (50%) (Qiagen, Germany) was added to cell lysates and incubated at room temperature (RT) for 2–3 h with continuous rotation. The beads were pelleted and supernatant was discarded. Beads were then washed two times with buffer I for 5 m in each step at RT followed by washing two times with buffer II (8 m Urea, 0.1 m Na_2_HPO_4_/NaH_2_PO_4_ pH 6.3, 150 mm NaCl, 0.2% Triton X-100, 20 mm imidazole, and 10 mm β-mercaptoethanol). His-tagged ubiquitinated proteins were eluted by incubating the beads in 50 μl of elution buffer (200 mm imidazole, 0.15 m Tris–HCl, pH 6.7, 30% glycerol, 0.72 m β-mercaptoethanol, 0 5% SDS) for 20 m at RT and was analyzed by Western blotting.

### *In vitro* ubiquitination assay

The ubiquitination assay of BACE1 was carried out using an ubiquitination kit from Enzo Life Science. For this, we have purified recombinant GST-BACE1 and His-CHIP from *E. coli* and ubiquitination reaction was carried out with 1.5 μL E1, 3 μL E2 (UbcH5a), 1.5 μL Mg-ATP buffer, 3 μL 10X ubiquitination buffer, 1.5 μL Ub, 2 μg GST-BACE1, 5 μg of His-CHIP, and H_2_O in a 30-μL volume at 30 °C for 1 h. The ubiquitinated BACE1 proteins were detected by Western blotting using the anti-BACE1 antibody.

### Chromatin immunoprecipitation (ChIP) assay

To fix the DNA–protein complex, cells were cross-linked with 1% formaldehyde at 37 °C for 10 m and then stopped by the adding 125 mm glycine. Cells was resuspended in 0.6 mL of lysis buffer (1% SDS, 10 mm EDTA, 50 mm Tris–HCl, pH 8.0, and cocktail protease inhibitor) and incubated for 15 m on ice. Sonication was carried out for 2 m (pulse on 5 s and pulse off 5 s) followed by centrifugation. The supernatant was diluted in dilution buffer (1% Triton X-100, 2 mm EDTA, 150 mm NaCl, 20 mm Tris–HCl, pH 8.0), and immunoprecipitation was carried out over night with p53 antibodies at 4 °C. Twenty-five microliters of protein A–Sepharose beads (saturated with salmon sperm DNA) was added and incubated on a rotary shaker for 2 h at 4 °C. Agarose beads were pelleted and washed sequentially for 10 m each in TSE I (0.1% SDS, 1% Triton X-100, 2.0 mm EDTA, 20 mm Tris–HCl, pH 8.0, and 150 mm NaCl), TSE II (0.1% SDS, 1% Triton X-100, 2.0 mm EDTA, 20 mm Tris–HCl, pH 8.0, and 500 mm NaCl) and buffer III (0.25 m LiCl, 1% NP-40, 1% Na deoxycholate, 1 mm EDTA, 10 mm Tris–HCl, pH 8.0). Beads were then washed once with TE buffer. The immunocomplex was eluted twice with 150 μl of elution buffer (1% SDS and 0.1 m NaHCO_3_) after 15 m incubation at RT. De-cross-linking was performed at 65 °C in a water bath. The DNA was extracted and PCR was carried out with appropriate primers. Equal amount of chromatin solution was precipitated with no antibody as a negative control.

### Real-time PCR

Quantitative PCRs were performed on an ABI 7000, using SYBR-Green Master Mix (Applied Biosystems), ABI PRISM® 96-well optical reaction plates and ABI PRISM™ optical adhesive plate sealers. All reactions were completed in triplicate. Each 20 μL PCR contained 0.02–0.1 μg cDNA, 2X SYBR-Green Master Mix, and primers diluted to a final concentration of 0.5 μm. The following cycling parameters were used: 50 °C for 10 min, then 95 °C for 10 min. This was followed by 40 cycles of 95 °C for 10 s and a combined annealing/extension temperature of 60 °C for 2 m. During each cycle of the PCR, the fluorescence emitted by the binding of SYBR-Green dye to the double-stranded DNA produced in the reaction was measured. To confirm the specificity of the reactions, dissociation curves were constructed for each primer pair at 0.1 °C intervals between the temperatures of 60 °C and 95 °C.

### Statistical analysis

Comparisons of the difference in mean of two groups (±SEM) were carried out using the Student’s *t*-test. Comparisons were two-tailed. All statistical tests were carried out using Sigma Plot, version 11 statistics software. *P *< 0.05 was accepted as significant. Mean values (±SEM) for each experiment with more than two groups were calculated using one-way ANOVA.
